# Methicillin-Resistant *Staphylococcus aureus* in the Food Chain: Molecular Epidemiology, Resistance Mechanisms, and Public Health Implications

**DOI:** 10.3390/ijms27093814

**Published:** 2026-04-24

**Authors:** Ayman Elbehiry, Adil Abalkhail, Ahmed Elnadif Elmanssury, Eman Marzouk

**Affiliations:** Department of Public Health, College of Applied Medical Sciences, Qassim University, P.O. Box 6666, Buraydah 51452, Saudi Arabia; ar.elbehiry@qu.edu.sa (A.E.);

**Keywords:** methicillin-resistant *Staphylococcus aureus*, antimicrobial resistance, molecular epidemiology, livestock-associated MRSA, virulence factors, biofilm formation, genomic surveillance, public health

## Abstract

Methicillin-resistant *Staphylococcus aureus* (MRSA) is a major antimicrobial-resistant pathogen affecting both human and animal health. Although historically associated with healthcare settings, MRSA is now established in livestock production and throughout the production chain. Its detection in animals, food products, and processing environments reflects the complex ecology of antimicrobial resistance (AMR) in modern food systems. This narrative review synthesizes current evidence on the molecular basis of methicillin resistance and multidrug resistance determinants, as well as the epidemiology of MRSA in food-associated settings. Particular emphasis is placed on its occurrence in animal-derived foods and key reservoirs within farms, slaughterhouses, and processing environments. Livestock-associated populations are dominated by clonal complex CC398. In contrast, CC9 is prevalent in pig production systems in Asia, while CC5-related lineages occur at the human and animal interface. MRSA has been detected in retail meat and animal-derived foods at low but measurable prevalence, indicating contamination during slaughter and processing. Virulence determinants include staphylococcal enterotoxins linked to food poisoning and Panton–Valentine leukocidin associated with severe infections. Biofilm formation and adhesins further support persistence and colonization. Epidemiological and molecular evidence indicates that livestock, processing environments, and food-contact surfaces act as interconnected reservoirs sustaining MRSA circulation. Human exposure occurs primarily through occupational contact and environmental pathways, whereas foodborne transmission appears less common. Effective control requires integrated surveillance, responsible antimicrobial use in livestock production, and strict hygiene practices throughout the production chain within a One Health framework.

## 1. Introduction

Antimicrobial resistance (AMR) is a major global public health challenge [1,2]. Since their introduction, antibiotics have transformed the treatment of infectious diseases [3]. However, their widespread and often inappropriate use in human medicine, veterinary practice, and agriculture has accelerated the emergence of resistant microorganisms [4,5]. As a result, many infections are becoming increasingly difficult to treat in both hospital and community settings [6]. Global estimates indicate that bacterial AMR was directly responsible for approximately 1.27 million deaths and associated with nearly 4.95 million deaths worldwide in 2019 [7].

Among bacterial pathogens, *Staphylococcus aureus* (*S. aureus*) is a major opportunistic organism affecting humans and animals. It causes a broad range of diseases, including skin and soft tissue infections, bloodstream infections, pneumonia, and endocarditis [8]. Its adaptability and ability to acquire resistance genes through mutation and horizontal gene transfer have driven the emergence of multidrug-resistant strains [9].

The emergence of methicillin-resistant *S. aureus* (MRSA) represents a key step in the evolution of AMR. First reported shortly after the introduction of methicillin in the 1960s, MRSA demonstrates the rapid response of this pathogen to antibiotic pressure [10]. Resistance is primarily mediated by acquisition of the *mecA* gene, which encodes PBP2a, a penicillin-binding protein with reduced affinity for β-lactam antibiotics [11,12]. This mechanism allows continued cell wall synthesis in the presence of these drugs, resulting in resistance to most β-lactams and contributing to the persistence of MRSA as a major cause of difficult-to-treat infections worldwide [8,9,13].

Although initially associated with healthcare settings, MRSA has expanded beyond clinical environments. Increasing evidence illustrates its presence in livestock production systems and along the food supply chain. Food-producing animals, including pigs, cattle, and poultry, act as reservoirs, contributing to the emergence of livestock-associated MRSA (LA-MRSA) lineages [14,15]. These strains may spread through direct animal contact or contamination during slaughter and processing [15].

MRSA has also been detected in various food products, including raw meat, poultry, and processed foods [15,16]. While foodborne transmission appears less common than direct exposure, contamination of food products indicates a potential route for dissemination of resistant strains [16].

The occurrence of MRSA in food production systems results from interactions between animals, food products, processing environments, and humans within a One Health framework [17,18]. Understanding these interconnected pathways requires integration of molecular resistance mechanisms, clonal distribution, and transmission dynamics [11,19].

Despite extensive research, knowledge remains fragmented across clinical, veterinary, and food safety disciplines. Many studies address resistance mechanisms or epidemiology separately, without integrating these components within the context of the food system.

This review provides an integrated overview of MRSA from a One Health perspective, combining current knowledge on resistance mechanisms, genomic epidemiology, and occurrence within the food chain. The aim is to clarify how resistant strains emerge, persist, and spread across interconnected environments and to identify key gaps for future research.

## 2. Literature Search Strategy

Relevant studies were identified through systematic searches of PubMed, Web of Science, and Scopus, covering publications from 1995 to early 2026. This period was selected because it corresponds to the widespread adoption of molecular approaches for characterizing *S. aureus*, including detection of the *mecA* gene and early typing methods.

Search terms included “methicillin-resistant *Staphylococcus aureus* (MRSA)”, “antimicrobial resistance (AMR)”, “livestock-associated MRSA (LA-MRSA)”, “mecA”, “SCCmec”, “food chain”, “food contamination”, and “molecular epidemiology”. These terms were applied individually and in combination to ensure comprehensive coverage.

Only peer-reviewed articles published in English were included. Studies were selected if they addressed resistance mechanisms, virulence determinants, molecular epidemiology, or the occurrence and transmission of MRSA in livestock, food products, or food-processing environments. Studies lacking clear methodological description or direct relevance were excluded.

The search identified 1398 records, of which 1124 remained after duplicate removal. After title and abstract screening, 242 full-text articles were assessed, and 79 primary studies were included in the qualitative synthesis. Selection was based on relevance, methodological quality, and contribution to the topic, with priority given to recent studies while retaining key earlier work. This review follows a narrative approach; therefore, no formal quantitative selection framework was applied.

## 3. Evolutionary Emergence and Epidemiological Lineages of MRSA

### 3.1. Emergence of Methicillin Resistance in S. aureus

MRSA was identified shortly after the introduction of methicillin in 1959, with resistant isolates reported within two years in the United Kingdom [20,21]. Resistance is primarily mediated by acquisition of the *mecA* gene, which encodes PBP2a, an altered penicillin-binding protein that allows cell wall synthesis to continue in the presence of β-lactam antibiotics [21,22].

The *mecA* gene is carried on the staphylococcal cassette chromosome mec (SCCmec), a mobile genetic element that facilitates horizontal transfer between strains [23].

SCCmec elements are classified into several types based on their genetic structure. Larger types (I–III) are typically associated with hospital-associated MRSA (HA-MRSA) and often carry additional resistance genes, whereas smaller types (IV and V) are more common in community-associated MRSA (CA-MRSA) [22,23].

### 3.2. Major Epidemiological Lineages of MRSA

MRSA has diversified into distinct populations adapted to different environments and is commonly grouped into HA-MRSA, CA-MRSA, and LA-MRSA lineages [21].

HA-MRSA represents the earliest group and remains a major cause of healthcare-associated infections, often showing resistance to multiple antimicrobial classes and carrying larger SCCmec elements [13]. Prominent clones include ST239, ST5, and ST22 [13,24].

CA-MRSA emerged later and is typically associated with infections in individuals without healthcare exposure. These strains often carry smaller SCCmec elements and may harbor virulence factors such as Panton–Valentine leukocidin (PVL). Representative lineages include ST8 (USA300), ST30, and ST80 [21,25]. Prominent CA lineages include ST8 (USA300), as well as ST30 and ST80, reported in different regions [13,25].

LA-MRSA is linked to food-producing animals and agricultural environments. These strains have been identified in pigs, cattle, and poultry, as well as in individuals with occupational exposure [26,27]. The most widely stated lineage is clonal complex 398 (CC398), which is prevalent in livestock populations and is transmitted between animals and humans [27].

### 3.3. Adaptation of MRSA to Livestock Reservoirs

LA-MRSA represents a key link between animal and human reservoirs. Its emergence was recognized in the early 2000s following detection in pigs and in individuals working in livestock production [28]. Most isolates belong to CC398, which is widely distributed in livestock populations [18,28].

Genomic evidence suggests that this lineage originated from a human-associated ancestor and subsequently adapted to animal hosts through acquisition of resistance determinants and genetic changes supporting persistence in livestock environments [27].

Livestock production systems provide conditions that facilitate colonization and spread. High carriage rates have been reported in pigs, and individuals in contact with animals display increased colonization [29].

Although CC398 predominates in many regions, other lineages such as CC9 have been reported in livestock, particularly in Asia, indicating that multiple lineages adapt to animal hosts [15,30].

**Figure 1:**
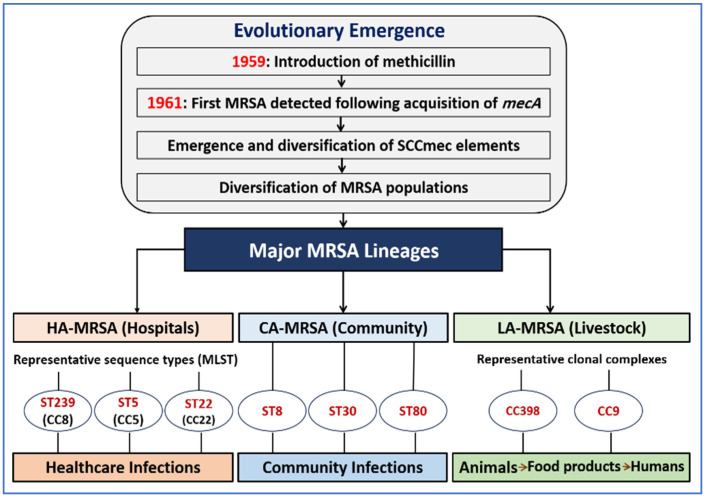
Evolutionary emergence and diversification of MRSA lineages. Following the introduction of methicillin, *S. aureus* acquired the mecA gene within the SCCmec element, leading to the emergence of MRSA. Subsequent diversification produced major epidemiological lineages, HA-MRSA, CA-MRSA, and LA-MRSA each linked to distinct ecological reservoirs. The figure highlights lineage divergence and their association with clinical, community, and livestock environments, including potential dissemination within the supply chain.

The presence of MRSA in livestock also raises concerns regarding entry into the food chain during slaughter and processing [31]. The evolutionary emergence and diversification of MRSA lineages are illustrated in Figure 1.

## 4. Molecular Basis of Methicillin Resistance

### 4.1. The mec Gene Complex

Methicillin resistance in *S. aureus* is driven by acquisition of the mec gene complex, most notably *mecA*, which encodes PBP2a. This alternative penicillin-binding protein sustains peptidoglycan synthesis despite β-lactam-mediated inhibition of native PBPs [13,32,33,34,35].

Expression of *mecA* is regulated by *mecI* and *mecR1*, which respond to β-lactam exposure. Disruption of this regulatory system, commonly observed in MRSA, leads to constitutive resistance [36,37,38].

An alternative determinant, *mecC*, has been identified in both human and animal isolates, indicating circulation across different reservoirs [39,40].

### 4.2. Structure and Function of PBP2a

PBP2a sustains cell wall synthesis under β-lactam pressure by retaining transpeptidase activity despite antibiotic exposure. Its low affinity for β-lactams allows continued peptidoglycan cross-linking when native PBPs are inhibited [11,41,42].

Structural studies have identified an allosteric site that regulates access to the catalytic domain, enabling substrate binding while limiting antibiotic interference [43].

### 4.3. Staphylococcal Cassette Chromosome mec (SCCmec)

The mec gene complex is carried on SCCmec, a mobile genetic element that integrates into the chromosome of *S. aureus* and enables horizontal transfer of resistance [44,45].

**Figure 2:**
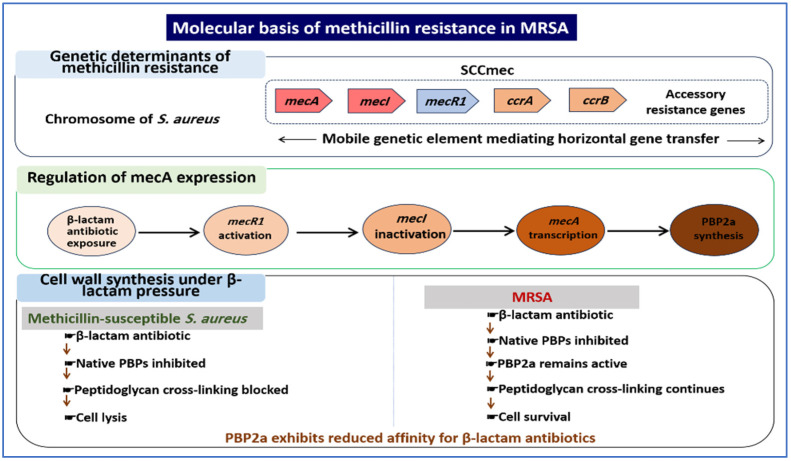
Molecular mechanism of methicillin resistance in MRSA. The SCCmec element integrates into the chromosome of *S. aureus* and carries the *mecA* gene encoding PBP2a. In the presence of β-lactam antibiotics, the MecR1–MecI regulatory system induces mecA expression, leading to production of PBP2a, which maintains peptidoglycan cross-linking despite inhibition of native penicillin-binding proteins.

SCCmec contains both the mec complex and cassette chromosome recombinase (*ccr*) genes, which mediate integration and excision. Variability in these elements defines distinct SCCmec types that differ in size and genetic content, with some carrying additional resistance determinants [23,45,46,47]. The organization of SCCmec and its role in resistance are illustrated in Figure 2.

### 4.4. Auxiliary Resistance Factors

Additional genes involved in cell wall synthesis, including members of the *fem* family, contribute to full resistance expression by supporting the peptidoglycan structure [9,47,48,49]. Global regulatory systems further influence resistance levels, contributing to phenotypic variability among MRSA strains [9,50].

### 4.5. Evolution of SCCmec Elements

SCCmec diversity is associated with adaptation to different ecological niches. Larger elements are typically linked to HA-MRSA and often carry multiple resistance genes, whereas smaller elements are more common in community-associated strains [51,52,53]. This diversity supports the spread of MRSA between clinical, animal, and environmental settings [51].

## 5. Multidrug Resistance Determinants in MRSA

MRSA commonly exhibits resistance to multiple antimicrobial classes beyond β-lactams. This phenotype arises from a combination of acquired resistance genes and chromosomal mutations affecting antibiotic targets or intracellular drug accumulation [54,55].

### 5.1. Macrolides and Lincosamides

Resistance is mainly mediated by *erm* genes, which encode methylases that modify 23S rRNA and prevent antibiotic binding [56,57]. Common variants (*ermA*, *ermB*, and *ermC*) are often located on mobile elements [58,59].

Inducible resistance may occur upon antibiotic exposure, while efflux systems such as *msrA* reduce intracellular drug concentrations [60,61].

### 5.2. Tetracycline

Resistance involves efflux and ribosomal protection mechanisms. *tetK* encodes an efflux pump, whereas *tetM* protects the ribosome by displacing tetracycline from its binding site [56,62,63,64]. These genes are widely disseminated between human and animal populations.

### 5.3. Fluoroquinolone

Resistance arises from mutations in genes encoding DNA gyrase and topoisomerase IV. Alterations in *gyrA/gyrB* and *grlA/grlB* reduce drug binding, with combined mutations leading to higher resistance levels [65,66,67,68,69,70,71,72].

### 5.4. Efflux Systems and Environmental Selection

Efflux systems contribute to multidrug resistance by lowering intracellular antibiotic concentrations [63,73]. Transporters such as NorA, NorB, NorC, MdeA, LmrS, and MepA act on diverse compounds [74,75,76], and their overexpression is associated with increased resistance [77,78].

Plasmid-encoded pumps such as *qacA* and *qacB* confer tolerance to disinfectants, particularly quaternary ammonium compounds, linking resistance mechanisms to food-processing environments [79,80]. Exposure to sublethal disinfectant concentrations may select for strains carrying these genes and promote persistence [81,82].

The accumulation of multiple resistance determinants enhances survival under antimicrobial pressure and supports the multidrug-resistant phenotype observed in MRSA [55,63]. Their distribution across lineages facilitates dissemination between clinical settings, animal reservoirs, and food production systems [14,83].

In food-related environments, selective pressures extend beyond antibiotic use to include disinfectants and other stress conditions, shaping resistance profiles and persistence along the production continuum [81,82]. Major resistance determinants are summarized in Table 1.

## 6. MRSA Reservoirs in the Food Production Continuum

The food production continuum includes multiple environments that support MRSA persistence. Resistant strains occur between livestock, slaughterhouses, and processing facilities, forming linked reservoirs within production systems [14,16].

### 6.1. Livestock Reservoirs

Food-producing animals represent major reservoirs of MRSA, with reports in pigs, cattle, poultry, and other species worldwide [14]. Colonization occurs mainly in the nasal cavity and on the skin and is often asymptomatic. Pigs are particularly important reservoirs and are frequently associated with CC398 lineages [89,90].

Cattle and poultry also harbor MRSA, indicating that multiple species contribute to its maintenance in agricultural systems [14]. Colonization persists within farms and spreads through direct contact and shared environments. Individuals in close contact with livestock, including farmers and veterinarians, show higher carriage rates than the general population [91]. MRSA has also been detected in airborne dust and bioaerosols within livestock facilities [92,93].

### 6.2. Slaughterhouse and Processing Environments

Slaughterhouses represent a key interface between animal reservoirs and food production. During processing, bacteria from colonized animals contaminate carcasses, equipment, and surrounding surfaces [16].

MRSA has been identified on carcasses, cutting surfaces, and processing equipment, indicating contamination throughout slaughter operations [31,94]. In swine production systems, strains present in animals have the ability to enter processing environments during handling.

Cross-contamination occur at multiple stages, including skin removal, cutting, and equipment handling, allowing transfer between carcasses and surfaces [16].

### 6.3. Food Handling and Processing Surfaces

Food-processing environments provide conditions that support MRSA persistence. *S. aureus* survives on stainless steel and plastic commonly used as food-contact surfaces [95].

Persistence is influenced by moisture and organic residues, which promote surface conditioning and increase the likelihood of contamination [95,96,97]. Biofilm formation further enhances survival by protecting cells from environmental stress and reducing the effectiveness of cleaning procedures [96].

Monitoring studies have detected *S. aureus* on surfaces within meat-processing facilities, particularly during active production [97]. Biofilms enable prolonged surface attachment, and cells released from these structures contaminate food products during processing [98].

**Figure 3:**
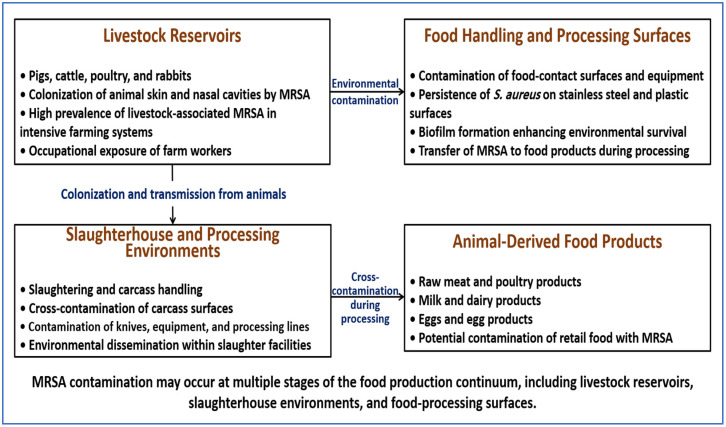
MRSA reservoirs and contamination routes among the food production continuum. Schematic representation of linked reservoirs of MRSA, including livestock, slaughterhouse environments, and food-processing surfaces. During slaughter and processing, bacteria from colonized animals contaminate carcasses, equipment, and contact surfaces, enabling cross-contamination within production systems. These processes facilitate the presence of MRSA in animal-derived food products such as meat, milk, and eggs, highlighting the role of the food production continuum as a pathway for dissemination.

Persistence in these environments results from repeated contamination and adaptation to local conditions. Surface adhesion, biofilm formation, and tolerance to disinfectants enable certain strains to persist despite routine sanitation. The role of biofilm formation is discussed in Section 9.3. Major reservoirs and contamination pathways are illustrated in Figure 3.

## 7. Occurrence of MRSA in Food Products

MRSA has been reported in a range of food products across regions, with contamination identified in retail and production settings, including raw meat, dairy products, and ready-to-eat (RTE) foods. Entry into the food chain occur during animal production, slaughter, processing, or handling [99,100,101].

Detection in food does not imply transmission through consumption but indicates contamination at different stages and highlights the need to monitor antimicrobial-resistant bacteria in food systems. These data clarify movement of resistant strains between animals, food products, and humans [19,102]. Representative findings are summarized in Table 2.

### 7.1. Raw Animal-Derived Food Products

Raw animal-derived foods are among the most studied sources of MRSA contamination. MRSA has been detected in retail meat, including beef, pork, and poultry, with evidence linking these strains to livestock reservoirs and contamination during slaughter or processing [99,103]. In the Netherlands, MRSA was found in 11.9% of retail meat samples (264/2217), with higher rates in poultry [99].

Studies from North America report lower but variable detection rates. In the United States, MRSA was identified in 6.6% (9/136) and 1.2% (2/165) of retail meat samples in separate investigations [100,104]. Genetic similarity between isolates from meat and livestock supports contamination during production stages.

MRSA has also been detected in milk and dairy products. Sources include infected animals, particularly mastitis cases, and contamination during milking or processing [105,106]. In Iran, MRSA was identified in ~2.0% of samples (53/2650), representing 16.2% of *S. aureus* isolates [106]. In Italy, prevalence was lower at 0.37% (6/1634) in animal-derived foods [103].

### 7.2. RTE Foods

RTE foods present a potential exposure route because they are consumed without further heat treatment. Contamination may occur during preparation, packaging, or handling by colonized workers [101]. Reported prevalence is generally lower than in raw meat. In China, MRSA was detected in 2.5% (5/197) of retail food samples, corresponding to 12.2% of *S. aureus* isolates [105]. Despite a lower frequency, the absence of a final decontamination step makes even low-level contamination relevant [101].

### 7.3. Global Distribution and Prevalence

MRSA contamination of food products has been reported worldwide, with prevalence varying by region, food type, and study design. Sample-based estimates range from low to moderate levels. Reported rates include 11.9% in retail meat in the Netherlands (264/2217) [99], 6.6% (9/136) and 1.2% (2/165) in the United States [100,104], 2.5% (5/197) in retail foods in China [105], and approximately 2.0% (53/2650) in dairy products in Iran [106]. Lower values have been described in some settings, such as 0.37% (6/1634) in Italy [103].

Variation is driven by differences in sampling strategies, food categories, and analytical methods. Some studies report MRSA as a proportion of *S. aureus* isolates rather than total samples, which complicates comparison. For example, MRSA accounted for 7.4% of *S. aureus* isolates in retail meat in China [101].

Across studies, prevalence typically ranges from <1% in certain dairy products to >10% in some meat samples. These differences likely reflect both regional factors and methodological variation, including detection approaches and reporting criteria [99,100,101,102,103,104,105,106]. Livestock production practices, antimicrobial use, and hygiene conditions during processing further influence these estimates [107].

Consistent detection across settings suggests repeated introduction from animal reservoirs rather than sustained growth within food products. This suggests that food acts primarily as a transient carrier rather than a stable reservoir [99,100,101,102,103,104,105,106,107,108].

Most studies report detection frequencies rather than standardized prevalence, limiting direct comparison across datasets. Although systematic reviews confirm the presence of MRSA in retail food products across multiple countries, lack of harmonized surveillance remains a key limitation. Standardized approaches are needed to improve comparability and interpretation of global data [108].

Overall, available evidence shows consistent but generally low-level contamination of food products, supporting a limited role in direct transmission but a potential contribution to MRSA dissemination along the food chain.

## 8. Molecular Epidemiology of Foodborne MRSA

Molecular epidemiology provides a framework for characterizing the genetic diversity, transmission pathways, and evolutionary relationships of MRSA populations in animal production systems and along the food chain [109,110]. Typing methods enable comparison of isolates from livestock, food products, and humans, supporting identification of transmission links and clonal structure across regions and environments [47,111].

MRSA populations consist of several genetically distinct lineages that are differentiated using sequence-based methods, including multilocus sequence typing (MLST), staphylococcal protein A (*spa*) typing, SCCmec typing, and pulsed-field gel electrophoresis (PFGE) [47,112,113].

### 8.1. Classical Molecular Typing Methods

Classical typing methods have been central to defining MRSA population structure and tracking dissemination among human, animal, and food sources [114,115]. Standardized protocols enable consistent data generation and contribute to global surveillance of MRSA dissemination [116,117].

MLST characterizes isolates based on sequence variation in conserved housekeeping genes, assigning sequence types that are grouped into clonal complexes representing evolutionary lineages [47,118,119,120]. This approach has been widely used to describe the global distribution of major MRSA clones [14,47].

*Spa* typing targets the polymorphic X region of the *spa* gene, generating repeat-based profiles that allow rapid differentiation of isolates. Its simplicity and standardized nomenclature make it suitable for surveillance and outbreak investigations [121,122]. SCCmec typing classifies isolates according to the structure of the cassette carrying the mec gene complex, providing insight into lineage origin and association with hospital, community, or livestock settings [123,124].

PFGE provides high-resolution discrimination based on restriction patterns of chromosomal DNA, enabling detection of closely related isolates. However, its limited reproducibility and labor-intensive workflow have reduced its use in favor of sequence-based approaches [113,125,126,127].

### 8.2. Whole-Genome Sequencing (WGS) in MRSA Surveillance

WGS enables high-resolution analysis of MRSA genomes, allowing direct comparison between strains and precise characterization of genetic variation [128,129,130,131].

Genome-level analysis identifies single nucleotide polymorphisms (SNPs), mobile genetic elements, resistance genes, and virulence determinants, supporting reconstruction of phylogenetic relationships and lineage evolution [132,133,134].

Recent studies have applied WGS to compare isolates from livestock, food products, and processing environments. Analyses across multiple stages of the production chain including slaughterhouse and retail settings allow assessment of genetic relatedness between isolates recovered at different points [135,136].

For example, isolates from livestock, slaughterhouse environments, and retail meat have been shown to cluster within closely related lineages, supporting epidemiological links across the production continuum [137,138]. These isolates often differ by fewer than 20 SNPs, a range consistent with recent transmission rather than independent emergence [27,138].

WGS enhances resolution among closely related strains and reveals transmission links not resolved by conventional typing methods [132,133,134]. It also supports differentiation between persistent contamination within processing environments and repeated introduction from animal reservoirs [135,136].

**Figure 4:**
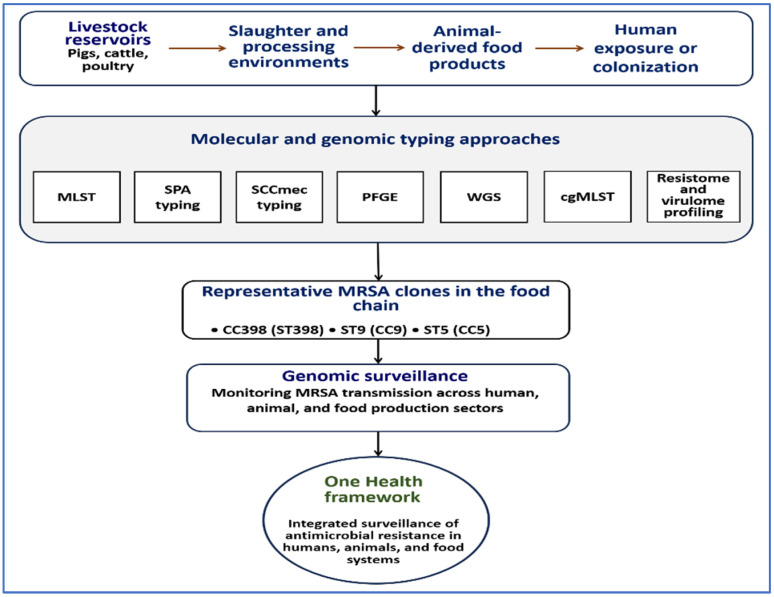
Molecular and genomic approaches for investigating MRSA dissemination along the food production continuum. The diagram integrates transmission pathways of MRSA from livestock reservoirs through slaughter and processing environments to animal-derived food products, together with analytical approaches used to characterize these processes. Classical typing methods (MLST, *spa* typing, SCCmec typing, and PFGE) and genomic approaches, including WGS, SNP-based phylogeny, and core genome MLST (cgMLST), enable high-resolution analysis of clonal relationships, resistance determinants, and virulence profiles. These tools support identification of transmission links and surveillance of MRSA across animal, food, and human sectors within a One Health framework.

Furthermore, WGS enables comprehensive analysis of resistance and virulence determinants, providing insight into adaptation of MRSA populations within food production systems [107,139,140,141,142]. These capabilities make WGS a central tool for modern AMR surveillance. The main molecular and genomic methods used to investigate MRSA transmission along the food chain are summarized in Figure 4.

### 8.3. Dominant MRSA Clones in the Food Chain

Several MRSA clones are associated with livestock and food production systems [143], as summarized in Table 3.

CC398 is the most widely distributed lineage in food-producing animals and represents the predominant livestock-associated MRSA clone [14,144,145,146]. Within livestock populations, ST398 strains how genetic diversity and are often associated with *spa* types such as t011 and t034. These strains commonly colonize animals and may infect humans with occupational exposure, indicating zoonotic potential [147].

ST9 is frequently detected in pig production systems, particularly in Asia, and has been identified in both animals and pork products, indicating circulation within agricultural environments and entry into the food chain [148,149].

Human-associated clones, including ST5, have also been detected in food products, suggesting exchange between clinical and food production settings [150,151].

The presence of multiple lineages between animals, food products, and humans illustrates the complexity of MRSA epidemiology in interconnected systems [26]. Continued molecular surveillance, particularly using genomic approaches, remains essential for detecting emerging clones and clarifying transmission patterns [152].

**Table 3:** Major MRSA clones detected in livestock and food production systems.

Clone/Lineage	Primary Host	Geographic Distribution	Typical Spa Types	Common SCCmec Types	Key References
CC398 (ST398)	Pigs, cattle, poultry	Europe, North America, Asia	t011, t034	IV, V	[26,90,145]
ST9 (CC9)	Pigs	East and Southeast Asia	t899 lineage variants	IV, V	[148,149]
ST5 (CC5)	Humans, livestock	Global	t002 and related types	II, IV	[47,150]
ST8 (CC8)	Humans; occasionally food products	North America	t008	IV	[47,100]

## 9. Virulence Determinants of Foodborne MRSA

Virulence determinants shape the pathogenic potential of MRSA in food-associated contexts. These include toxins, cytolytic proteins, and surface-associated factors that mediate colonization, immune evasion, and persistence in production environments [153,154]. Key traits relevant to foodborne MRSA include staphylococcal enterotoxins, PVL, and mechanisms supporting biofilm formation and surface attachment [155,156].

### 9.1. Staphylococcal Enterotoxins

Staphylococcal enterotoxins are major contributors to foodborne illness caused by *S. aureus* [157,158]. These toxins are heat-stable and remain active after food processing, leading to rapid-onset symptoms including nausea, vomiting, abdominal cramps, and diarrhea following ingestion of contaminated food [159,160].

Classical enterotoxins (SEA–SEE) are most frequently associated with outbreaks, although additional variants have been identified in food-related isolates. These toxins act as superantigens, triggering widespread T-cell activation and excessive cytokine release that underlies gastrointestinal symptoms [161].

Enterotoxin-encoding genes such as *sea*, *seb*, *sec*, and *sed* have been detected in MRSA isolates from food products, indicating the presence of toxin-producing strains [162].

### 9.2. Panton–Valentine Leukocidin (PVL)

PVL is a cytolytic toxin associated with severe *S. aureus* infections. It consists of LukS-PV and LukF-PV components that form pores in host immune cells, particularly neutrophils and macrophages [163,164,165].

PVL is commonly linked to CA-MRSA and has been associated with skin infections and necrotizing pneumonia. Its genes are associated with specific lineages, supporting their role in virulence [166,167,168,169].

Although primarily described in clinical isolates, PVL has also been identified in strains from animals and food products, indicating that food-associated MRSA may carry clinically relevant virulence traits [170].

### 9.3. Biofilm Formation and Persistence

Biofilm formation plays a central role in MRSA persistence in food production environments. These structured communities are embedded in a matrix that protects cells from environmental stress, disinfectants, and host defenses [171]. In food-processing settings, development is influenced by surface properties and environmental conditions. Materials such as stainless steel and plastic support attachment, while organic residues enhance surface conditioning and promote biofilm establishment [171,172,173,174,175,176,177].

Substrates including raw meat, protein-rich residues, and dairy components further support adhesion and biofilm development. Organic matter forms conditioning layers rich in proteins and lipids that modify surface properties and facilitate attachment. MRSA isolates from food sources form biofilms on materials such as stainless steel and polystyrene, with protein-rich matrices contributing to structural stability [178,179]. Similar observations in dairy environments highlight persistence in the presence of food residues [95,180]. These conditions support survival on equipment surfaces and increase cross-contamination risk [181,182].

Biofilm formation is supported by genes such as *icaA* and *icaD*, which mediate synthesis of polysaccharide intercellular adhesin and stabilize biofilm structure [172]. The presence of these determinants in isolates from food and processing environments enhances survival under routine conditions, while temperature variation and sanitation practices further influence biofilm stability [162].

### 9.4. Surface Adhesins and Host Colonization

Surface adhesins enable MRSA to attach to host tissues and abiotic surfaces. These proteins bind extracellular matrix components such as fibronectin, fibrinogen, and collagen, facilitating early stages of colonization [154,173].

Key adhesin genes include *clfA*, *clfB*, *fnbA*, *fnbB*, and *cna*, which contribute to adherence and persistence [174,175]. These genes have also been identified in isolates from food and processing environments, indicating an enhanced capacity for surface attachment and persistence during production [176,177].

These virulence determinants contribute to MRSA survival and dissemination along the food chain. Enterotoxins are directly linked to foodborne illness, whereas biofilm formation and adhesins support persistence and contamination in production environments, influencing potential exposure to humans [154,174].

## 10. Transmission Pathways from the Food Chain to Humans

MRSA transmission between animals, food products, environments, and humans occurs within the structure of modern food production systems. These pathways link animal health, food safety, and human exposure within a One Health framework [151,183,184,185].

Epidemiological evidence reveals that MRSA spreads within these interfaces through direct contact, environmental exposure, and contaminated food products, enabling movement between ecological niches and contributing to the wider dissemination of AMR [186,187].

### 10.1. Occupational Exposure

Occupational contact represents a primary route linking livestock reservoirs to human carriage. Individuals working in animal production, including farmers, veterinarians, and slaughterhouse workers, are frequently exposed to colonized animals and contaminated environments through direct contact, inhalation of dust, or exposure to animal secretions [186,188].

Higher MRSA carriage rates have consistently been reported among livestock workers compared with the general population. Colonization commonly occurs in the nasal cavity and may persist depending on exposure intensity. Livestock-associated lineages such as CC398 have been identified in these individuals, supporting their zoonotic potential [26,188].

### 10.2. Foodborne Exposure

Food products derived from colonized animals represent an additional route of human exposure. Contamination occurs during slaughter, processing, or handling, facilitating the transfer of bacteria from animals or processing environments to products that reach consumers [16,151].

Although infection through consumption appears less frequent than direct exposure, detection of MRSA in food products confirms that resistant strains enter the food supply. Handling raw products or consuming inadequately prepared foods may result in transient colonization or exposure to enterotoxin-producing strains [189]. Molecular evidence showing similarity between isolates from food and livestock supports the role of contaminated products in dissemination [14,189].

### 10.3. Environmental Dissemination

Environmental reservoirs contribute to indirect transmission between animals and humans. MRSA shed by colonized animals persists in farm environments, slaughter facilities, and processing areas, remaining on surfaces or within organic material [190,191].

Airborne dust, bioaerosols, and contaminated surfaces act as carriers in agricultural settings. Viable MRSA has been recovered from airborne particles in livestock facilities, indicating potential for distribution within and beyond these environments [192,193].

Contamination of processing environments further supports persistence and cross-contamination through equipment and contact surfaces, reinforcing the role of environmental reservoirs in transmission along the food chain [194].

## 11. Public Health Implications

The presence of MRSA in food production systems represents a continuing public health concern. Resistant strains have been detected in animals, food products, and production environments, indicating their ability to persist and move between sectors [16,195,196].

Human exposure occurs through occupational, environmental, and food-related pathways, with higher colonization rates observed in individuals working in livestock settings [91,189,195,197]. Although the risk associated with food consumption appears limited, contaminated products may introduce resistant bacteria into the community [189].

A broader concern is the dissemination of AMR. MRSA strains in production environments frequently harbor multiple resistance determinants, driving spread across bacterial populations and complicating infection control in clinical settings [14,47,198].

Addressing this issue requires coordinated surveillance, effective hygiene practices, and responsible antimicrobial use among animal production systems.

## 12. Surveillance and Control Strategies

Limiting MRSA circulation in food production systems requires coordinated action between human, animal, and environmental sectors. Movement of resistant bacteria between livestock, food products, and human populations makes isolated interventions insufficient. Effective control depends on integrated measures combining surveillance, responsible antimicrobial use, and consistent hygiene practices along the production chain [199,200].

### 12.1. Integrated One Health Surveillance Systems

Integrated surveillance is essential for monitoring AMR. The One Health approach supports coordinated tracking of resistant bacteria across human, animal, food, and environmental sectors, improving early detection of emerging resistance patterns and clarifying transmission pathways [201,202].

These systems integrate data from clinical laboratories, veterinary monitoring programs, food inspection networks, and environmental sampling. This allows identification of resistant strains in animal populations, detection of contamination in production systems, and assessment of risks to human health [203].

Several countries have implemented national monitoring programs based on this model, generating data that support risk assessment and guide evidence-based policies to limit AMR spread [204].

### 12.2. Antimicrobial Stewardship in Livestock Production

Responsible antimicrobial use in livestock production is critical for reducing the emergence and spread of resistance. Stewardship programs promote appropriate antibiotic use through guidelines addressing drug selection, dosing, and treatment duration [205].

In some systems, antibiotics have been used for disease prevention and growth promotion, creating selective pressure that favors multidrug-resistant bacteria, including MRSA [200,206].

Stewardship efforts therefore focus on reducing non-therapeutic use and promoting alternative strategies such as vaccination, improved animal management, and strengthened biosecurity. These measures help maintain animal health while limiting selective pressure in livestock populations [207].

### 12.3. Hygiene and Food Safety Interventions

Hygiene measures throughout the production chain are essential for limiting contamination. These include farm biosecurity, sanitation of equipment in slaughter facilities, and strict hygiene during processing and handling [208].

Cleaning and disinfection reduce bacterial load and limit cross-contamination between animals, equipment, and food products. Consistent application is critical; otherwise, inadequate sanitation or the introduction of contaminated animals leads to persistence within production systems [208].

Additional measures include hazard analysis and critical control point systems, routine microbiological monitoring, and training of food handlers. These approaches reduce contamination risk and help prevent entry of resistant organisms into the food supply [209].

## 13. Future Perspectives and Research Priorities

Future research should focus on strengthening surveillance and improving understanding of MRSA transmission between human, animal, and environmental reservoirs. Advances in molecular epidemiology have improved characterization of MRSA populations and enabled identification of emerging lineages, but continued expansion of monitoring programs remains necessary to track their spread [169,210].

A key priority is the broader application of genomic surveillance. WGS enables detailed analysis of MRSA evolution, resistance determinants, and transmission pathways. Integrating genomic data with epidemiological and environmental information enhances detection of emerging lineages, supports risk assessment, and informs targeted control measures within a One Health framework [17,211,212,213,214].

Metagenomic approaches provide complementary insight by enabling detection of AMR genes directly from environmental and food samples without prior isolation. These methods expand understanding of the resistome in agricultural settings and may support earlier identification of emerging resistance [211].

Further research is required to clarify MRSA ecology in livestock systems, including mechanisms of persistence, host adaptation, and environmental survival. Studies integrating genomic, ecological, and epidemiological data will help define factors that sustain resistant strains in agricultural environments [169].

Development of rapid diagnostic technologies represents another important direction. Advances in molecular and culture-independent methods may enable faster detection of MRSA in clinical, food, and environmental samples, supporting earlier intervention and improved surveillance [212].

Finally, interdisciplinary collaboration remains essential. Integration of data in human health, veterinary, food safety, and environmental sectors is required to better understand MRSA circulation and to develop effective strategies for limiting the spread of antimicrobial resistance through the food chain [210].

## 14. Conclusions

MRSA is no longer confined to healthcare settings and is now established across livestock production systems and the food supply chain. Its presence in animals, processing environments, and food products arises from linked sources that enable transfer of resistant strains between sectors. Molecular and genomic analyses have clarified the diversity of MRSA lineages and their dissemination between animal, food, and human interfaces, reinforcing the need for coordinated surveillance. Virulence determinants, including toxins and surface-associated factors, support persistence in production environments and contribute to pathogenic potential. Although foodborne infection appears less common than direct exposure routes, contaminated products and environments may still facilitate human contact with resistant strains. Limiting MRSA dissemination requires integrated surveillance, responsible antimicrobial use in livestock, and effective hygiene practices during processing and handling, supported by coordinated, system-level action across sectors.

## Figures and Tables

**Table 1:** Major AMR genes identified in MRSA and their associated antibiotic classes.

Antibiotic Class	Major Resistance Genes	Mechanism of Resistance	References
Macrolides/Lincosamides	*ermA*, *ermB*, *ermC*	Ribosomal methylation of 23S rRNA preventing binding of MLSB antibiotics	[84,85]
Macrolides/Streptogramins	*msrA*	ATP-binding cassette efflux transporter exporting macrolides	[85]
Tetracyclines	*tetK*	Membrane efflux pump reducing intracellular tetracycline concentration	[86]
	*tetM*	Ribosomal protection protein displacing tetracycline from the ribosome	[86]
Fluoroquinolones	*gyrA*/*gyrB* mutations	Target modification of DNA gyrase	[87]
	*grlA*/*grlB* mutations	Target modification of topoisomerase IV	[87]
Multidrug resistance	*norA*	Major facilitator superfamily (MFS) efflux pump exporting multiple antibiotics	[72]
	*norB*, *norC*	Multidrug efflux transporters contributing to drug efflux	[72]
	*mepA*	MATE family multidrug efflux pump	[88]
Antiseptics/disinfectants	*qacA*, *qacB*	Plasmid-encoded efflux pumps conferring tolerance to quaternary ammonium compounds	[81,82]

**Table 2:** Reported occurrence of MRSA in food products from different regions of the world.

Food Product	Country/Region	Main Finding	Reference
Animal-derived foods	Italy	MRSA detected in 0.37% (6/1634)	[103]
Retail meat	Netherlands	MRSA detected in 11.9% (264/2217)	[99]
	United States	MRSA detected in 6.6% (9/136)	[100]
		MRSA detected in 1.2% (2/165)	[104]
Retail meat (isolates)	China	MRSA detected in 7.4% of *S. aureus* isolates (48/648)	[101]
Retail foods		MRSA detected in 2.5% (5/197)	[105]
Raw milk & dairy	Iran	MRSA detected in ~2.0% (53/2650)	[106]

## Data Availability

No new data were created or analyzed in this study.
